# Variability in Diagnosing Brain Death at an Academic Medical Center

**DOI:** 10.1155/2017/6017958

**Published:** 2017-03-02

**Authors:** Ashutosh Pandey, Pradeep Sahota, Premkumar Nattanmai, Christopher R. Newey

**Affiliations:** Department of Neurology, University of Missouri, 5 Hospital Drive, CE 540, Columbia, MO 65211, USA

## Abstract

*Objective*. Research continues to highlight variability in hospital policy and documentation of brain death. The aim of our study was to characterize how strictly new guidelines of American Academy of Neurology (AAN) for death by neurological criteria were practiced in our hospital prior to appointment of neurointensivists.* Method*. This is a retrospective study of adults diagnosed as brain dead from 2011 to 2015. Descriptive statistics compared five categories: preclinical testing, neurological examination, apnea tests, ancillary test, and documentation of time of death. Strict adherence to AAN guidelines for brain death determination was determined.* Result*. 76 patients were included in this study. Preclinical prerequisites were fulfilled in 53.9% and complete neurological examinations were documented in 76.3%. Apnea test was completed in 39.5%. Ancillary test was completed in 29.8%. Accurate documentation of time of death occurred in 59.2%. Overall, strict adherence to current AAN guidelines for death by neurological criteria was correctly documented in 38.2%.* Conclusion*. Our study shows wide variability in diagnosing brain death. These findings led us to update our death by neurological criteria policy and increase awareness of brain death determination with the goal of improving our documentation following current AAN guidelines.

## 1. Introduction

Devastating brain injuries are well-recognized causes leading to irreversible cessation of neurological function and eventual brain death [1]. The Uniform Determination of Death Act (UDDA) in 1981 legally established brain death as “irreversible cessation of all functions of the entire brain, including the brain stem” [2]. The American Academy of Neurology Practice Parameter (AANPP) in 1995 published guidelines on how to determine brain death in the adult patient [3]. These guidelines were revised in 2010 to provide clearer instructions on determining brain death after a study in 2008 showed that many hospital policies were loosely adherent to the original 1995 guidelines in brain death determination [4, 5]. Despite the efforts of the AAN, studies continue to show wide variability in hospital policies for documentation requirements of brain death determination [6, 7].

Recently, neurointensivists were appointed at our tertiary care center. They were asked to review the current brain death policy. Neurointensivists have specialized training in managing critically ill neurosciences patient but also have expertise in the examination and prognosticating in devastating brain injuries [7]. The purpose of our retrospective study was to review variability in brain death diagnoses based on the hospital protocol prior to the appointment of the neurointensivists and identify areas for improvement.

## 2. Methods

We retrospectively reviewed the electronic medical record (EMR; Cerner, North Kansas City, MO) of 76 adult patients (age ≥ 18 years) diagnosed as having brain death from 2011 to 2015 at an academic tertiary care center. For the purpose of this study, we strictly limited our review of documentation to the brain death determination/death note. The project was exempt from approval by the Institutional Board Review because all patients were deceased.

Deidentified demographic data were extracted from the EMR including age, sex, and race. We then reviewed the brain death determination data (i.e., the brain death and/or death note) for each patient based on five categories: preclinical testing, neurological examination, apnea test, ancillary test, and documentation of time of death.

For preclinical testing, we looked at documentation for mechanism of death, absence of hypothermia (temperature ≤ 36°C), absence of hypotension (systolic blood pressure ≤ 100 mmHg), absence of confounding medical conditions (e.g., hyperammonemia, severe acidosis defined as pH < 7.2, thyroid abnormalities, and severe hypernatremia (>160 meq/dL)), and absence of drug/sedatives.

We reviewed the neurological examination for the following components: coma, absence of pupillary reflex, absence of corneal reflex, absence of both oculocephalic and oculovestibular reflexes, absence of both cough and gag, and absence of motor response to pain. We then reviewed the apnea test for completeness including documenting prerequisites (temperature ≥ 36°C, systolic blood pressure ≥ 100 mmHg, PaCO2 40 ± 5 mmHg, and PaO2 ≥ 90 mmHg and euvolemia), procedural details (e.g., amount of oxygen supplied in L/min and absence of respiratory effort), and the final PaCO2. If the apnea test was aborted, the reasons for the same were then reviewed for documentation.

The brain death note was reviewed for ancillary test, if performed. The note was reviewed for the type of ancillary test used either transcranial Doppler (TCD), electroencephalography (EEG), cerebral angiography, nuclear single-photon emission computed tomography (SPECT) scan, or other (e.g., computed tomography angiography (CTA), magnetic resonance imaging angiography (MRA), magnetic resonance imaging of brain (MRI brain)). Finally, notation was made of the service performing the brain death test and documentation of time of death.

Summary statistics were created to illustrate variability in brain death diagnosing and adherence of the current AAN guidelines.

## 3. Results

### 3.1. Patient Demographics

There were a total 76 brain death notes during the study period. The mean age was 43.78 ± 19.3. The majority were males (63.2%) and Caucasian (73.7%). The mechanism of injury causing the devastating brain injury included intracerebral hemorrhage (ICH; 34.2%), anoxia (31.6%), trauma (23.7%), and ischemic stroke (10.5%) (Table 1). Documentation of an irreversible neurological injury was documented in 40.8% of the specified brain death notes.

### 3.2. Physician Specialty

Neurologists determined the brain death in 13 (17.1%), neurosurgeons in 21 (27.6%), general surgeons/trauma surgeons in 27 (35.5%), and internists in 15 (19.7%) patients.

### 3.3. Prerequisites for Clinical Testing

**Figure 1:**
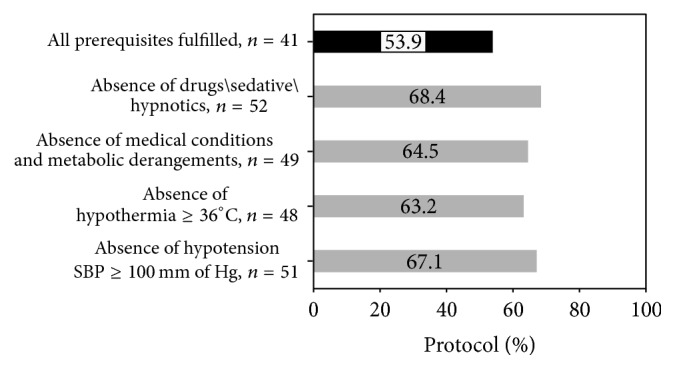
Prerequisites for clinical testing. Preclinical prerequisites: absence of drugs/sedative/hypnotics, absence of medical conditions and metabolic derangements, absence of hypothermia temperature ≥ 36°C, and absence of hypotension (SBP ≤ 100 mm of hg). SBP, systolic blood pressure; mmHg, millimeters of mercury; °C, degrees Celsius; *n*, number.

Fifty-two (68.4%) had absence of drugs/sedative or hypnotics. Forty-nine (64.4%) had absence of medical conditions and metabolic derangements. Prerequisites of absence of temperature ≤ 36°C and absence of hypotension (i.e., systolic blood pressure ≥100 mm of Hg) were fulfilled in 48 (64.5%) and 51 (67.1%), respectively. All four preclinical prerequisites were documented in 41 (53.9%; Figure 1).

### 3.4. Neurological Examination

**Figure 2:**
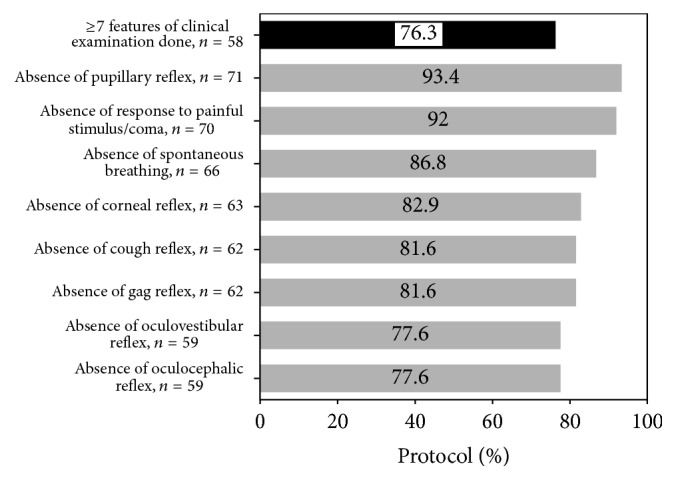
Specifics of clinical examination. Features of neurological examination: coma/absent response to painful stimulus, absent pupillary reflex, absent corneal reflex, absent cough reflex, absent gag reflex, absent oculovestibular reflex, and absent oculocephalic reflex. *n*, number.

Fifty (65.8%) patients had 2 examinations. The absence of pupillary reflex was noted in 71 (93.4%), absence of response to painful stimulus/coma in 70 (92%), absence of spontaneous breathing in 66 (86.8%), absence of corneal reflex in 63 (82.9%), absence of cough reflex in 63 (81.6%), absence of gag reflex in 62 (81.6%), absence of oculovestibular reflex in 59 (77.6%), and absence of oculocephalic reflex in 59 (77.6%; Figure 2).

Fifty-eight (76.31%) patients had all 7 features of neurological examination (including motor response to pain and brain stem reflexes, excluding absence of spontaneous breathing) documented as absent as per AAN guidelines (Figure 2). Eighteen (23.9%) had documented < 7 features of neurological examination. Out of these 18, 5 did not have any clinical features documented in the specified brain death note.

Forty (52.6%) patients had complete documentation of all 4 preclinical prerequisites and all 7 features of clinical neurological examinations.

### 3.5. Apnea Test

**Figure 3:**
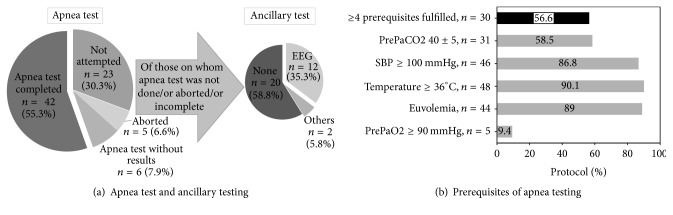
Apnea testing. Apnea test prerequisites: eucapnia, normotension, normothermia, euvolemia, and absence of hypoxia. EEG, electroencephalography; *n*, number; SBP, systolic blood pressure; °C, degrees Celsius; mmHg, millimeters of mercury; PaCO2, partial pressure of carbon dioxide; PaO2, partial pressure of oxygen; *n*, number.

Out of the 76 patients, apnea test was not attempted in 23 (30.3%). Apnea test was completed with result documentation in 42 (55.3%) patients. The result of apnea test was not documented in 6 (7.9%). Apnea test was aborted in 5 (6.6%; Figure 3(a)). In 2 patients, the apnea test was aborted due to hemodynamic instability and in 3 patients due to hypoxia.

#### 3.5.1. Apnea Test Prerequisites

Forty-four (83%) patients were euvolemic. Normothermia with core temperature of ≥36°C was in 48 (90.6%). 46 (86.8%) had normotension with systolic blood pressure of ≥100 mmHg prior to apnea test. Baseline PaCO2 prerequisite (40 ± 5 mm of Hg) prior to apnea test was fulfilled in 31 (58.9%) patients. Baseline PaO2 prerequisite (≥90 mm of Hg) prior to apnea test was fulfilled in 5 (9.4%) patients (Figure 3(b)).

#### 3.5.2. Apnea Test Results

Absence of respiratory effort documented in 43 (81.1%) and post-PaCO2 ≥ 60 mm of Hg or 20 mm of Hg above prePaCO2 was fulfilled in 42 (79.2%).

#### 3.5.3. Apnea Test Conclusion

Out of 42 on whom apnea test was done with result documentation, 30 (71.4%) had ≥4 prerequisites fulfilled. Three prerequisites were fulfilled in 9 (21.4%). Less than three prerequisites were fulfilled in 3 (7.1%) with 1 (2.3%) having no prerequisites fulfilled.

Twenty-nine (38.2%) patients fulfilled all 4 preclinical prerequisites, all 7 features of neurological examinations, and apnea test following ≥4 prerequisites as per 2010 AANPP guidelines.

### 3.6. Ancillary Testing

**Figure 4:**
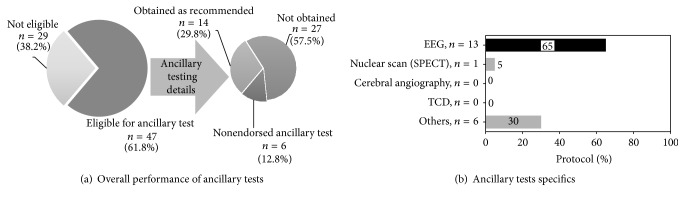
Ancillary tests. EEG, electroencephalograph; TCD, transcranial Doppler; SPECT, single-photon emission computed tomography; *n*, number.

Overall, 47 (63.5%) patients were eligible for ancillary testing. Out of these 47, 20 had ancillary testing, but only 14 (29.8%) had AAN approved ancillary tests (EEG in 13 and nuclear SPECT scan in 1). Six (12.8%) patients had other testing such as CT/A (*n* = 3) and MRI/A (*n* = 3). Twenty-seven (57.5%) patients who were eligible for ancillary testing did not have one (Figure 4). An additional two patients had ancillary testing who had already met brain death criteria prior to testing. These patients had an EEG (*n* = 1) and cerebral angiography (*n* = 1).

Twenty-one patients out of 35, for whom preclinical prerequisites were not fulfilled, did not have ancillary test (11 had EEG, 1 had nuclear scan, and 2 had others). Eight out of 11, for whom absence of all 7 features of clinical examination were documented but apnea test was not attempted, also did not have ancillary testing. Out of 18 patients with absence of <7 features of neurological examinations documented, 5 (27.8%) had EEG, 2 (11.1%) had others, 1 (5.6%) had nuclear (SPECT) scan, and 9 (50%) did not have any ancillary testing. Two out of 3 for whom absence of 4 to 6 reflexes were documented did not have ancillary testing. Seven of 15 for whom <4 features of clinical examination were documented did not have a completed ancillary test.

Fifteen out of 23 who did not have apnea test did not have ancillary testing. Two out of 5 patients for whom apnea test was aborted did not have an ancillary test. Three out of 6 patients for whom results of apnea tests were not documented did not have ancillary testing. Eleven out of 12 patients for whom ≤3 prerequisites for doing apnea test were completed did not have any ancillary testing.

Out of 34, for whom apnea test results were not documented or aborted or not attempted, 20 (58.8%) did not have any ancillary testing done, 12 (35.3%) had EEG, and 2 (5.8%) had ancillary tests not recommended by AAN (Figure 3(a)).

### 3.7. Documentation of Time of Death as in Apnea Test or Ancillary Test

Time of death in apnea and ancillary test was documented in 61 (80.3%) patients, but it only matched with the time of death mentioned in the death summary in 45 (73.8%). Out of 76, it was not applicable in 15 (19.7%) patients as they did not have either apnea or ancillary test.

### 3.8. Overall Adherence to AANPP 2010 Guidelines

**Figure 5:**
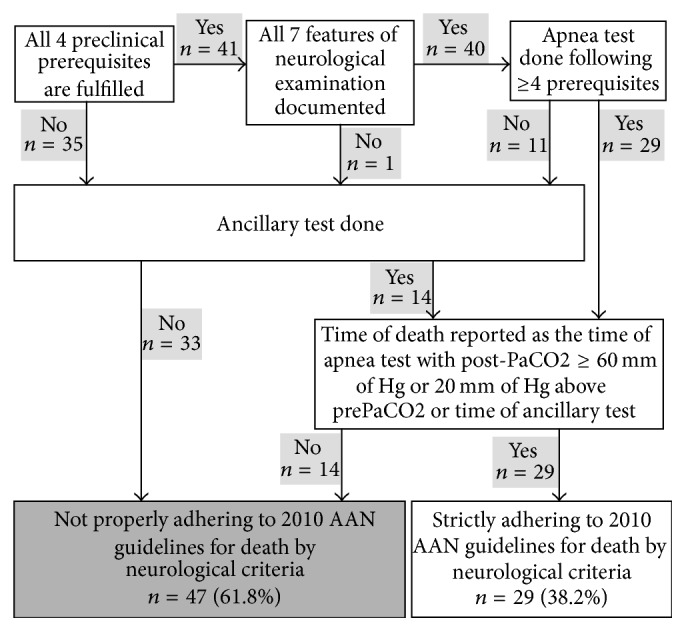
Overall adherence to AAN guidelines for death by neurological criteria. Preclinical prerequisites: absence of drugs/sedative/hypnotics, absence of medical conditions and metabolic derangements, absence of hypothermia, and absence of hypotension. Neurological examination: coma/absent response to painful stimulus, absent pupillary reflex, absent corneal reflex, absent cough reflex, absent gag reflex, absent oculovestibular reflex, and absent oculocephalic reflex. Apnea test prerequisites: eucapnia, normotension, normothermia, euvolemia, and absence of hypoxia. Ancillary test: electroencephalograph, single-photon emission computed tomography, transcranial Doppler, and cerebral angiography. *n*, number; AAN, American Academy of Neurology.

We found that documentation in 29 (38.2%) patients was strictly adherent to the 2010 AANPP guidelines for brain death determination. In 47 (61.8%) patients, 2010 AANPP guidelines were incompletely documented in the determination of brain death (Figure 5). Thirty-nine (51.3%) patients underwent organ donation. Twenty-three of these patients strictly adhered to the 2010 AANPP guidelines.

## 4. Discussion

Our study showed wide variability in diagnosing brain death at a single, academic center. Strict adherence to the AAN guidelines on diagnosing brain death was documented in 38.2% of patients. The variability was attributed to problems of the current policy as well as incomplete documentation. 55.2% of patients were diagnosed as having brain death by nonneuroscience services (i.e., internists or trauma surgeons). Only 44.7% of patients were diagnosed as having brain death by either neurology or neurosurgery.

The cardinal features of diagnosing brain death include (1) coma/absence of cerebrally mediated response to painful stimulus, (2) absent brainstem reflexes, (3) apnea test, and (4) documentation [5]. Despite this simplistic description, there is much variability [6, 7]. Shappell et al. (2013) reviewed charts from 68 hospitals [6]. They identified 226 brain dead organ donors of whom only 44.7% “strictly adhered” to the AAN guidelines on diagnosing brain death [6]. Importantly, only 45.1% had documented brainstem areflexia and absent motor response [6]. This group found 73.5% had completed apnea testing [6]. In our single center of 76 patients, we found that 76.3% of patients had documented brainstem areflexia and absent motor response but completed apnea test with documentation in only 55.3%. In those 23 patients who did not have apnea test attempted, 15 did not have ancillary testing. This is in contrast to the 99.3% in Shappell et al.'s study that showed ancillary testing was completed in those without apnea tests [6]. Our criteria for “strict adherence” is different from Shappell et al.'s [6], as we included two more features of current AAN guidelines while assessing the adherence. We included (1) fulfillment of prerequisites needed for doing apnea test and (2) documentation of time of death reported as the time of apnea test with post-PaCO2 ≥60 mm of Hg or 20 mm of Hg above prePaCO2 or time of interpretation of the ancillary test. We believe that these added features would have further decreased “strict adherence” in the study by Shappell et al. [6].

Greer et al. (2016) reviewed 492 hospital brain death policies for adoption of the AAN guidelines changes from 2010 [5, 7]. They found significant variability in the policies [7]. For example, 33.1% of policies required an expert in neurology or neurosurgery to diagnose brain death [7]. 65.9% of policies required two separate examinations [7]. Absence of hypotension as a preclinical prerequisite was only necessary in 56.2% of the policies [7]. The majority of policies required apnea test (97.4%) but only 83.5% specified a final PaCO2 value [7]. In 6.5% of the policies, ancillary testing was mandatory regardless of prior examination results [7]. Nonendorsed ancillary testing with either CTA or MRA was allowed in 9.0% and 2.9% of the policies, respectively [7]. These variabilities over brain death policies and documentations motivated us to review our policy in accordance with 2010 AANPP.

There are several disparities in our policy with current AAN guidelines.* First*, our policy allows any service/licensed physician to perform brain death testing and pronounce the patient as decided by primary care team. The lack of expertise in diagnosing brain death is a proposed source of error [7]. Trauma surgeons, who manage patients with severe traumatic brain injury in the setting of poly-system injuries, and medical internists diagnosed 55.2% of our brain death diagnoses. Neurologists and neurosurgeons completed only 44.7% of our brain death diagnoses.* Second*, policies requiring two examinations are not consistent with the updated guidelines. 65.8% of our patients had 2 examinations. Prolonging the diagnosis of brain death has been shown to have a negative impact on organ procurement [8]. In this study, as the interval time between brain death diagnoses increased, the refusal of organ donation increased from 23% to 36% [8].* Third*, an affirmative statement of irreversible brain injury that led to brain death was lacking. Certain clinical conditions can mimic brain death. For example, fulminant Guillain-Barre syndrome, high cervical cord injury, increased vecuronium due to delayed clearance, lidocaine or baclofen toxicity, and organophosphate intoxication [9–15]. Only 40.8% of our patients had documented mechanism of injury.* Fourth*, our current policy does not define hypothermia and shock in preclinical testing. AAN guidelines define these as systolic blood pressure ≥100 mm of Hg and temperature should be ≥36°C [5]. Only 46.1% of our patients had documented exclusion of confounding factors.* Fifth*, for neurological examination, the current policy allowed for the examiner to choose to perform either oculovestibular reflex or oculocephalic reflex. Recent guidelines state that absence of all brain stem reflexes should be documented [5]. 76.3% of our patients had documented absence of all 7 features of neurological examination. Additionally, the preclinical prerequisites are not fulfilled in 50% of these patients.* Sixth*, all prerequisites in preparation for the apnea tests were not mentioned as per guidelines [5]. In 56.6% of the patients, ≥4 prerequisites were fulfilled. In 23 (43.4%) patients, there were ≤3 prerequisites fulfilled prior to the apnea testing. In the eleven patients for whom the apnea test results were not documented or aborted, none of them had ≥4 prerequisites fulfilled.* Seventh*, our policy did not include TCD and cerebral angiography as accepted ancillary tests [5].

Nonendorsed ancillary tests such as CTA/MRA/MRI brain were not included in our policy. Nevertheless, CTA/MRA/MRI brain were completed in 27.3% as an “ancillary test.” Studies have shown sensitivity and divergent rate of CT/CTA are 52.4% and 30% respectively, when compared to angiography while diagnosing brain death [16, 17]. For MRI/MRA, although few studies have shown that brain death findings in MRI brain are very similar to the gold standard 4-vessel cerebral angiography, there are no confirmative studies which provide sufficient data to use MRI/A as an ancillary test [18, 19]. Fourteen patients had EEG, which is the most common ancillary test used at our center. Earlier studies have shown that EEG is affected by hypothermia, presence of drugs/sedatives/hypnotics, electrolytic or metabolic disorders, and artifacts [20, 21]. Moreover, EEG does not register the electrical activity of the lower brainstem. Furthermore, EEG may be flat when there is still cerebral blood flow and subcortical activity [22–24]. Nevertheless, AAN guidelines permit its use to identify brain death [5].

Our study showed that the variability in diagnosing brain death is also due to incomplete documentation. For example, 8 of 58 patients (for whom absence of all 7 features of clinical brain stem reflexes were documented) did not have apnea test and/or ancillary test. Five patients had no clinical features documented in the specified brain death note; 15 patients had ≤3 clinical features documented. Out of these 15, neither apnea test nor ancillary test was attempted in seven. Out of 76, 15 (19.7%) had neither apnea test nor ancillary test to determine brain death, and 1 (1.3%) patient did not have any documentation regarding clinical examination, apnea test, and/or ancillary test. However, it is possible that addressing confounders, mechanism of irreversible brain injury, and neurological examinations were documented elsewhere. Indeed, in the five cases without a clinical examination documented in the brain death and/or death note, the documentation was at least partially fulfilled in a progress note. Based on our criteria for this study, we limited our systematic review to the brain death determination and/or death note for complete documentation.

The variability in diagnosing brain death that we found in our hospital with respect to the 2010 AAN guidelines is a call to action. This study does not state that patients were inappropriately declared to be brain dead. As we mentioned, documentation may have been in progress notes not labeled as brain death notes and/or death notes. Rather, we sought to identify how to improve the variability in diagnosing and documenting brain death at a single, academic institution. We have since revised the death by neurological criteria protocol. It now includes detailed explanations of the brain death testing and a checklist based on the sample protocol provided by the Neurocritical Care Society [25]. Additionally, we have set forth our hospital guidelines to limit the diagnosis to more experienced physicians including intensivists, neurologists, neurosurgeons, and trauma surgeons. With only 76 cases of brain death in adults over the six-year period, it is reasonable for institutions to limit the diagnosis to experienced physicians. Additionally, we support the literature highlighting the benefits of simulation training in brain death [26].

Our study is limited by being retrospective. We chose the brain death note and death summaries as the sole sources for documentation of the brain death determination. As previously mentioned, documentation may have been in a routine progress note. However, this highlights the need for improved documentation. We also chose the time period, by design, as the hospital recently hired two board-certified neurointensivists who have since revised the hospital policy for diagnosing brain death. All patients in this study were diagnosed using the policy prior to the appointment of the neurointensivists. We will monitor for future progress in improved documentation with the updated protocol.

In conclusion, our study found wide variability in the documentation of brain death at our center. Accurate diagnosis of brain death includes a valid evaluation based on current standards along with a procedural protocol to ensure complete documentation. We have since updated our policy in accordance with the 2010 AAN guidelines and improved the documentation checklist. We do encourage other institutions to evaluate their policy and documentation.

## Figures and Tables

**Table 1:** Patient demographics.

Total patients	76
Age, y, mean (SD)	43.78 (19.3)
Sex, male, *n* (%)	48 (63.2%)
Race, *n* (%)	56 (73.7%)
White	6 (7.9%)
African American	2 (2.6%)
Hispanic	12 (15.8%)
Others	
Mechanism of death, *n* (%)	
Intracerebral hemorrhage	26 (34.2%)
Anoxia	24 (31.6%)
Trauma	18 (23.7%)
Stroke	8 (10.5%)

*n*, number; SD, standard deviation.
